# Anthocyanins Profile of Grape Berries of *Vitis amurensis*, Its Hybrids and Their Wines

**DOI:** 10.3390/ijms11052212

**Published:** 2010-05-21

**Authors:** Quan Zhao, Chang-Qing Duan, Jun Wang

**Affiliations:** 1 College of Forestry, Northeast Forestry University, Heilongjiang Province, Harbin 150040, China; 2 Center for Viticulture and Enology, College of Food Science & Nutritional Engineering, China Agricultural University, Beijing, 100083, China; E-Mail: chqduan@yahoo.com.cn; 3 Traditional Chinese Medicine Department, Jilin Agricultural Science and Technology College, Jilin Province, Jilin 132101, China; E-Mail: zhaoquanbs@163.com

**Keywords:** anthocyanins, *Vitis amurensis*, wine, HPLC-ESI-MS/MS

## Abstract

Anthocyanins are responsible for the color of grapes and wine, an important attribute of their quality. Many authors have used anthocyanins profile to classify the grape cultivars and wine authenticity. The anthocyanin profiles of grape berries of *Vitis amurensis*, its hybrids and their wines were analyzed by HPLC-ESI-MS/MS. The results identified 17 anthocyanins in these grape cultivars, including 11 anthocyanin monoglucosides (five pyranoanthocyanin monoglucosides and one acylated pyranoanthocyanin monoglucoside) and six anthocyanin diglucosides. Likewise, 15 kinds of anthocyanins were detected in wines, including six diglucosides and nine monoglucosides of anthocyanidins, in which four pyranoanthocyanin monoglucosides (Petunidin-3-*O*-glucoside-4-acetaldehyde, Malvidin-3-*O*-glucoside-4-pyruvic acid, Malvidin-3-*O*-glucoside-acetaldehyde and Peonidin-3-*O*-glucoside-4-pyruvic acid) were detected. In addition, a total of 14 kinds of anthocyanins including six diglucosides and eight monoglucosides of anthocyanidins were identified in skins, in which two pyranoanthocyanin monoglucosides (Peonidin-3-*O*-glucoside-4-pyruvic acid, Malvidin-3-*O*-glucoside-4-vinylphenol) and one acylated pyranoanthocyanin monoglucoside (Malvidin-3-*O*-(6-*O*-acetyl)-glucoside-4-vinylphenol) were detected. The anthocyanins profile of grape skin of *V. amurensis* and its hybrids consist of the anthocyanin monoglucosides, diglucosides and pyranoanthocyanins. The wines produced resulted in a slightly different anthocyanin distribution. Pelargonidin-3,5-diglucosides was first found in the skins and wines, however, no acetyl was detected in wines. The principal component analysis results suggest that the anthocyanin profiles were helpful to classify these cultivars of *V. amurensis*.

## 1. Introduction

Anthocyanins make up a group of pigments in grapes and wine, an important quality parameter that contributes an appealing color and antioxidant activity to red grapes and wine. Due to its bright color and high water solubility, this group is considered a potential natural pigment to replace artificial food colorants. On the other hand, this group also possesses potent antioxidant capacity and health promoting properties, even reducing the risk of cardiovascular diseases and some inflammatory diseases for people who consume wine, berries, and grapes [1–4].

The anthocyanins profile of a grape and its wine, determined by the relations of the different anthocyanins, is characteristic of each variety. An anthocyanin profile, or fingerprint, has also been used for many authors to differentiate the grape cultivars, and to identify the authenticity of red wines, especially if the grape cultivar is mentioned on bottle labels. Authors reported that the anthocyanin profiles provide enough information to develop a differentiation of classes in the studied wines or grapes [5–10].

With the development of HPLC-ESI-MS/MS and other determining techniques, more than 600 types of anthocyanins have been reported in nature. For grapes and wines, several hundreds of anthocyanins have been identified. These anthocyanins can be classified into the following groups according to their structure: non-acylated anthocyanins [common anthocyanin monoglucosides and diglucosides: monoglucosides of Cyanidin (Cy), Delphinidin (Dp), Petunidin (Pt), Peonidin (Pn), Malvidin (Mv)]; acylated anthocyanins, pyranoanthocyanins, direct flavanol-anthocyanin condensation products, acetaldehyde-mediated or other compound-mediated flavanol-anthocyanin condensation products, and polymeric anthocyanins [3,8,11–18]. The grapes of *V. vinifera* consist of only anthocyanin-monoglucoside [8,11–14,19], while *V. labrusca* and *V. rotundifolia* consist of not only anthocyanin monoglucoside but also anthocyanin diglucoside [3,8]. The wines which are made from these grapes (*V. vinifera, V. labrusca, V. rotundifolia*) also contain the corresponding anthocyanins [3,14,15]. The hybrid cultivars of Clinton (*V. lavrusca* × *V. riparia*) and Isabella (*V. vinifera* × *V. labrusca*) include anthocyanin monoglucosides and diglucosides [20].

*V. amurensis* is native to north-eastern China, and is resistant to low temperature, even at −40 °C. Wines made from these grapes have an unusual color, aroma and taste, quite different from the wine made from the grape *V. vinifera*. However, the anthocyanins profile of grape berries from *V. amurensis* and their wines is still not clear. In addition, it has not been sufficiently verified whether the grape and wine contains the pelargonidin anthocyanins, and whether the grape skin includes the oligomeric anthocyanins. The objective of this work is to identify the anthocyanin profile of grape berries of *V. amurensis* and further confirm the above problems by the HPLC-ESI-MS/MS analysis.

## 2. Materials and Methods

### 2.1. Analytical Standards and Reagents

Methanol was purchased from the Bei Jing Chemical Industry (China). Acetonitrile (HPLC grade), and formic acid (96%) was purchased from Fisher Scientific (Fairlawn, NJ, USA). Malvidin-3-glucoside chloride was purchased from Extrasynthese SA (Genay, France).

### 2.2. Samples

Grape berries of seven cultivars: SF (Shuang Feng, intraspecific hybrid of *V. amurensis*, hermaphroditic), SH (Shuang Hong, intraspecific hybrid of *V. amurensis*, hermaphroditic), SY (Shuang You, *V. amurensis*, hermaphroditic), Z1 (Zuo Shan Yi, *V. amurensis*, female, selected from wild resources), Z2 (Zuo Shan Er, *V. amurensis*, female, selected from wild resources), ZH (Zuo Hong Yi, interspecific hybrid, *V. amurensis* × Mycкat Poзabый×*V. amurensis*, hermaphroditic), ZY (Zuo You Hong, interspecific hybrid, *V. amurensis* × Mycкat Poзabый×*V. amurensis*, hermaphroditic) cultivated at the Institute of Special Wild Economic Animal and Plant, Chinese Academy of Agricultural Sciences and were sampled at maturation and harvest based on the color of grape berries. To obtain a sample representing a vineyard population, we sampled according to the method described by Boulton *et al*. [21]. Three 100-berry samples were selected from at least seven 10-cluster selections at similar positions of 30 whole vine selections. The fresh samples were kept in refrigerated bags, taken to the laboratory within a few hours, the skins peeled with forceps and frozen in liquid N_2_; then they were crushed as powder, which was frozen at −40 °C for anthocyanins extraction.

Wine samples were performed with a small glass container (10 L). The berries of seven cultivars for wine making were picked up at harvest. To each must, 50 mg/L SO_2_ was added before alcohol fermentation; then the activated yeast was added. After the fermentation was performed for four days, the pomace was separated from must and the last-fermentation was carried out. The wine samples were analyzed by HPLC-ESI-MS/MS with direct injection after filtration.

### 2.3. Extraction of Anthocyanins

The extraction of anthocyanins was performed according to Liang *et al*. with some modification [8]. 20 mL methanol with 5% (v/v) formic acid was added into 100 mL Erlenmeyer flasks that contained 1 g of grape skin powder. Anthocyanins were extracted at 30 °C for 30 min in a dark environment; this was repeated five times to collect the extract solution. The extraction was concentrated under vacuum at 30 °C using a rotary evaporator until dryness. The dry extraction was resolved in 5 mL solvent of 2% formic acid in distilled water. About 1 mL of extracted solution was strained through a 0.45 μm millipore filter for HPLC-ESI-MS/MS analysis.

### 2.4. HPLC-MS Analysis

An Agilent 1200 series LC-MSD, equipped with a UV detector and reversed phase column (Kromasil C18 250 × 4.65 μm), was used. The solvents were (A) aqueous 2% formic acid, and (B) acetonitrile containing 2% formic acid. The gradient was from 6% to 10% B for 4 min, from 10% to 25% B for 8 min, isocratic 25% B for 1 min, from 25% to 40% for 7 min, from 40% to 60% for 15 min, from 60% to 100% for 5 min, from 100% to 6% for 5 min, at a flow rate of 1.0 mL/min. Injection volumes were 30 μL, and the detection wavelength was 525 nm. Mass spectroscopy (MS) conditions were as follows: Electrospray ionization (ESI) interface, positive ion model, 35 psi nebulizer pressure, 10 L/min dry gas flow rate, 350 °C dry gas temperature, and scans at *m/z* 100–1000. All analyses were replicated twice.

### 2.5. Statistical Analysis

All individual anthocyanins were quantified and expressed as malvidin-3-glucoside content from the chromatographic results. If any of these anthocyanins remained undetected in a sample, they were represented by zero in the data matrix for principal component analysis (PCA). PCA was performed with the statistical software SPSS 15.0 (USA).

## 3. Results and Discussion

### 3.1. Anthocyanins in Grape Skins

**Figure 1.**
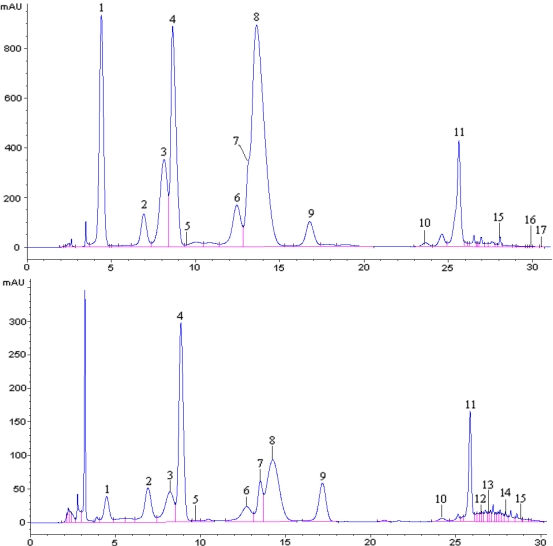
The chromatograms of the Zuo You Hong grape skin at harvest (upper) and the Zuo Hong Yi wine (lower).

There were 17 anthocyanins identified in *V. amurensis* grapes, their hybrids and their corresponding wines by HPLC-ESI-MS/MS (Table 1 and Figure 1).

**Figure 2.**
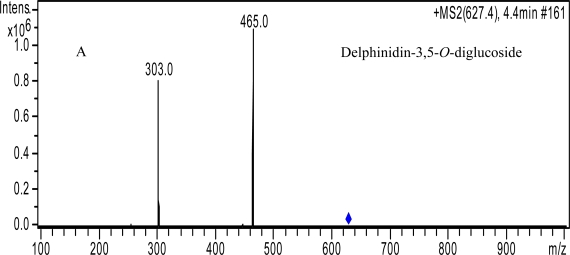

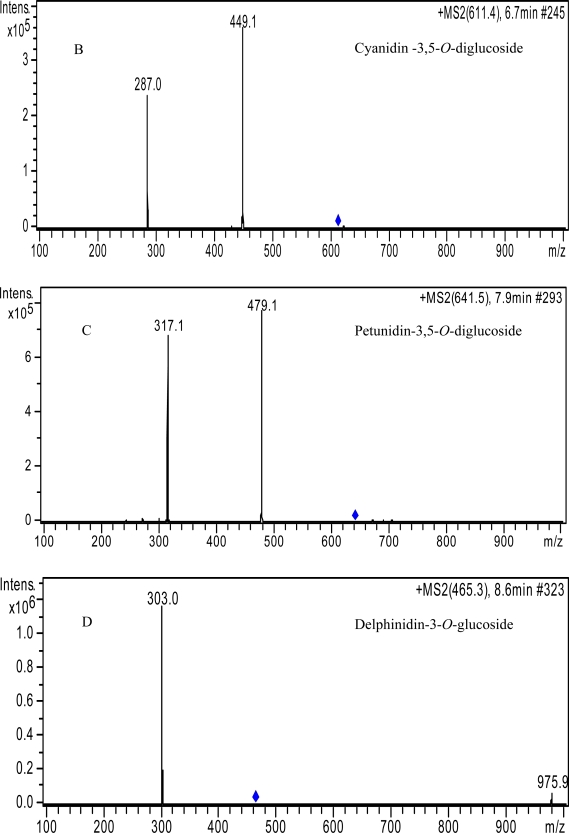

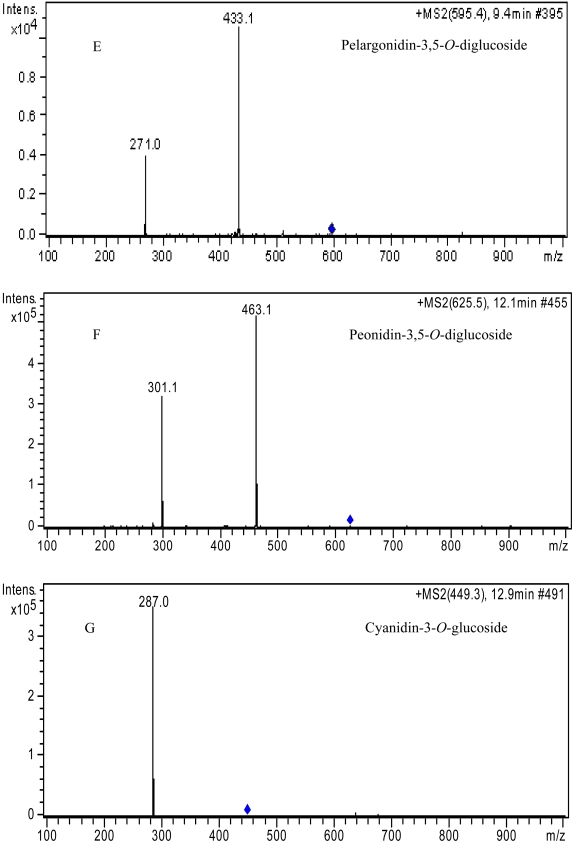

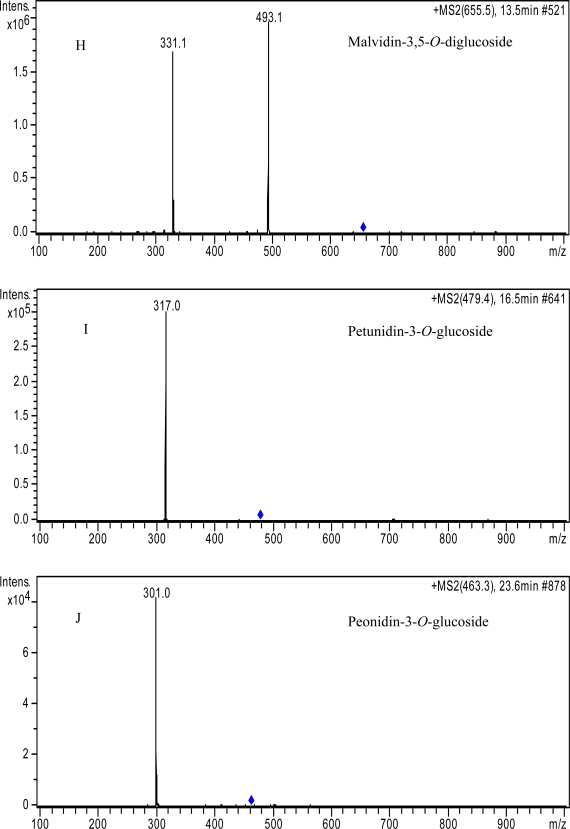

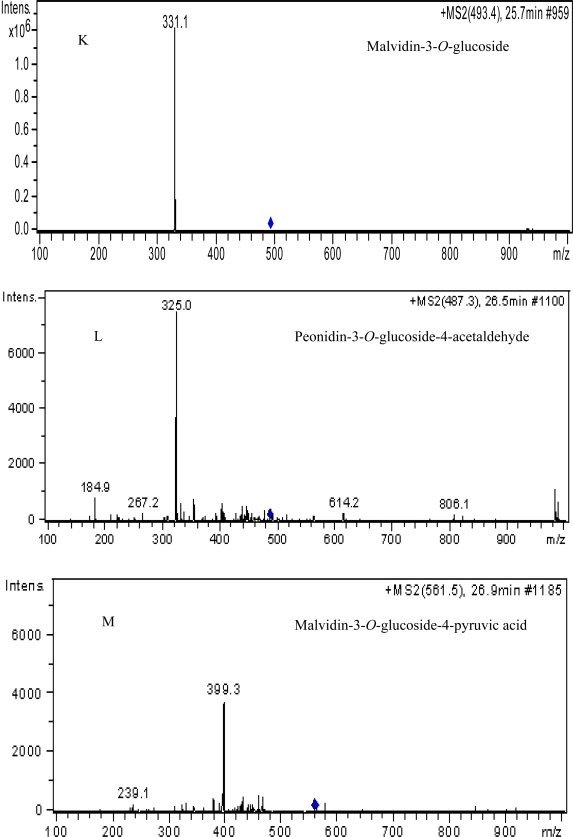

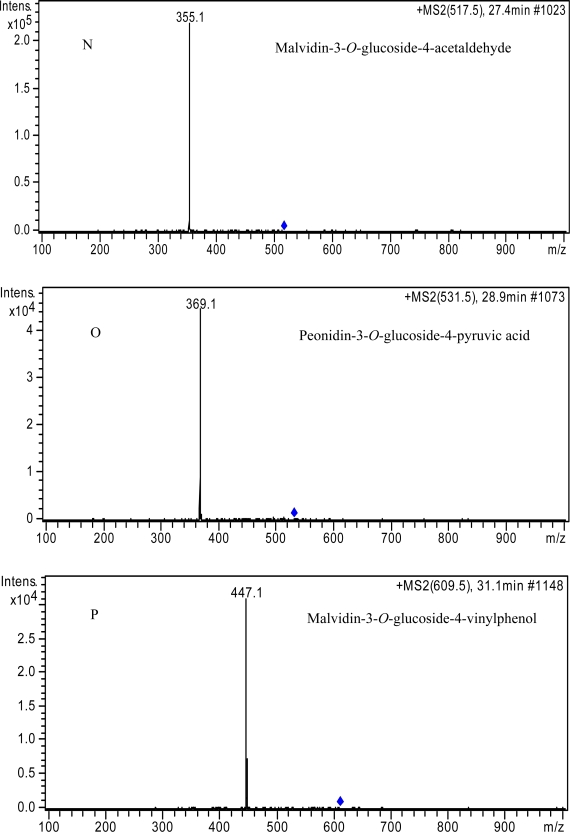

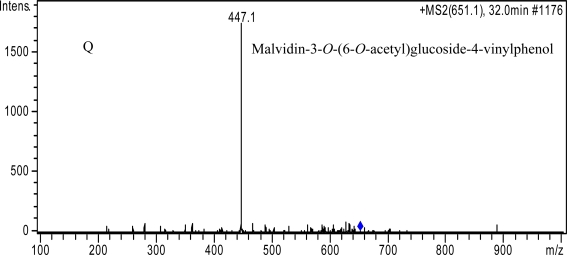
The molecular ion and ion fragments.

In grape of *V. amurensis*, the fragment model of peak No. 1 (*m/z* 627 (M^+^), 465, 303, Figure 2A), 2 (*m/z* 611 (M^+^), 449, 287, Figure 2B), 3 (*m/z* 627 (M^+^), 465, 303, Figure 2C), 5 (*m/z* 595 (M^+^), 433, 271, Figure 2E), 6 (*m/z* 625 (M^+^), 463, 301, Figure 2F), 8 (*m/z* 655 (M^+^), 493, 331, Figure 2H) were the same, all of which lost two glucose units (−162, −162) in sequence to product the corresponding fragment ions. They were identified as the six common anthocyanin diglucosides, delphinidin-3,5-*O*-diglucoside, cyanidin-3,5-*O*-diglucoside, pelargonidin-3,5-*O*-diglucoside, petunidin-3,5-*O*-diglucoside, peonidin-3,5-*O*-diglucoside, malvidin-3,5-*O*-diglucoside. The molecular and product ions of peak No. 5 was the compound pelargonidin-3,5-*O*-diglucoside [22]. Wang *et al*. also reported that pelargonidin-3-*O*-glucoside is present in the Concord (*V. labrusca*), Rubired (*V. vinifera* × *V. rupestris*), and Salvador (*V. vinifera* × *V. rupestris*) grape juices [23]. This is, to our knowledge, the first time that any research has reported the presence of pelargonidin-3,5-*O*-diglucoside in *V. amurensis* grape.

The molecular ions of peak No. 4 (*m/z* 465 (M^+^), 303, Figure 2D), 7 (*m/z* 449 (M^+^), 287, Figure 2G), 9 (*m/z* 479 (M^+^), 317, Figure 2I), 10 (*m/z* 463 (M^+^), 301, Figure 2J), 11 (*m/z* 493(M^+^), 331, Figure 2K) lost one glucose unit (−162) to produce the corresponding fragment ions of anthocyanidins (303, 287, 317, 301, 331). They were identified as the anthocyanin monoglucoside: delphinidin-3-*O*-glucoside, petunidin-3-*O*-glucoside, peonidin-3-*O*-glucoside, malvidin-3-*O*-glucoside, cyanidin-3-*O*-glucoside.

The molecular ion and fragment ion of peak No. 15 was 531(M^+^), 369 (Figure 2O), identified as peonidin-3-*O*-glucoside-4-pyruvic acid. While that of peak No. 16 was 609(M^+^), 447 (Figure 2P) and 17 was 651(M^+^), 447 (Figure 2Q), which were identified as malvidin-3-*O*-glucoside-4-vinylphenol and malvidin-3-*O*-(6-*O*-acetyl)-glucoside-4-vinylphenol, respectively.

In the grapes of cultivars SY, Z1 and Z2, which all belong to *V. amurensis*, HPLC-ESI-MS/MS detected that they comprise of 12 anthocyanins, although some anthocyanin was not detected in maturation. However, peonidin-3-*O*-glucoside-4-pyruvic acid was not detected in the varieties of SF and SH, which also belong to *V. amurensis*, and comprise of 11 anthocyanins.

The hybrid of ZH was comprised of the same anthocyanin with the *V. amurensis* (SY, Z1, Z2), but the hybrid of ZY also included the two other anthocyanins malvidin-3-*O*-glucoside-4-vinylphenol and malvidin-3-*O*-(6-*O*-acetyl)-glucoside-4-vinylphenol. Vidal *et al.* confirmed the existence of anthocyanin oligomers in the grape skin extract by mass spectrometric evidence [24].

In *V. vinifera* grapes, not only are the monoglucosides of delphinidin, cyanidin, petunidin, peonidin and malvidin present, but also their acetyl, coumaroyl and caffeoyl derivatives as well. While in wines made from the *V. vinifera* grapes, the monoglucosides of pyranoanthocyanins and other polymeric anthocyanins are also present. However, the grape of Pinot Noir (*V. vinifera*) contains only five common anthocyanin monoglucosides (delphinidin-glucoside, cyanidin-glucoside, petunidin-glucoside, peonidin-3-*O*-glucoside, and malvidin-3-*O*-glucoside) [25].

### 3.2. Anthocyanins in Wines

The presence of 15 anthocyanins in these seven wines made from the *V. amurensis* and its hybrids can be seen in Table 1. These wines consist of six anthocyanin diglucosides and nine anthoyanin monoglucosides (four pyranoanthocyanin monoglucosides).

The ZH comprised of 15 kinds of anthocyanins detected in all wines, while Peonidin-3-*O*-glucoside and petunidin-3-*O*-glucoside-4-acetaldehyde was not detected in the ZY wine. In the wines made from *V. amurensis,* petunidin-3-*O*-glucoside and malvidin-3-*O*-glucoside-4-acetaldehyde was not detected in the SF and SY wine, respectively. While peonidin-3-*O*-glucoside and malvidin-3-*O*-glucoside-4-pyruvic acid were not detected in the SH wine. Malvidin-3-*O*-glucoside-4-acetaldehyde was not detected in wine of Z2, while two pyranoanthocyanins (Malvidin-3-*O*-glucoside-4-pyruvic acid, Malvidin-3-*O*-glucoside-4-acetaldehyde) were not detected in the wine of Z1.

The anthocyanin profile of grape and wine detected two anthocyanins (Malvidin-3-*O*-glucoside-4-vinylphenol, Malvidin-3-*O*-(6-*O*-acetyl)-glucoside-4-vinylphenol) in the grape skins remained undetected by HPLC-ESI-MS/MS in their corresponding wines.

### 3.3. PCA Results of Grape Cultivars and Wines

Principal components analysis was also performed, obtaining that the first three components account for more than 80.0% of the total variance in all these analysis.

**Figure 3.**
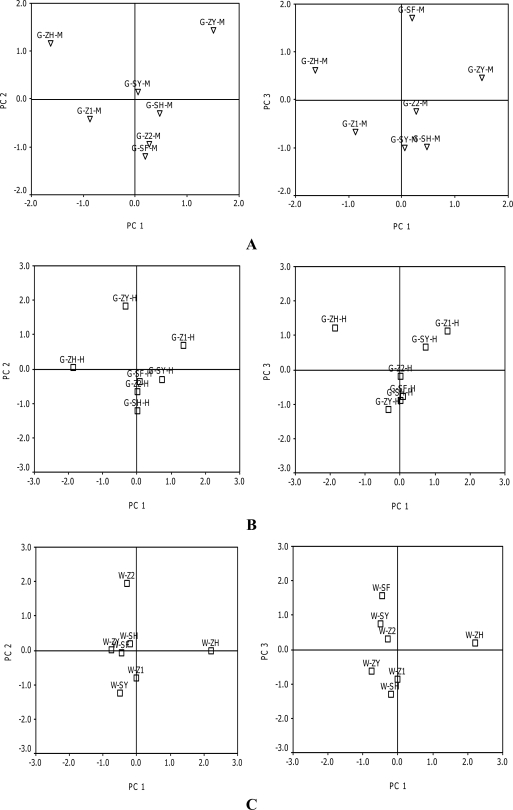
Principal components analysis of the anthocyanin profile of grapes and wines. **A:** grape at maturation (PC1 *vs*. PC2; PC1 *vs.* PC3). **B:** grape at harvest (PC1 *vs.* PC2; PC1 *vs.* PC3). **C:** wines (PC1 *vs.* PC2; PC1 *vs.* PC3) See Table 1 for definitions of abbreviations.

As it is shown in Figure 3A, the cultivar G-ZH-M and G-ZY-M stands out, which turns out to be clearly distanced from the rest and separated well by PC1. The cultivar G-SY-M, G-Z1-M, G-SH-M, G-Z2-M and G-SF-M were mainly separated along the PC2; however, G-SY-M, G-Z1-M, G-SH-M could not be distanced enough even though they were located in a different quadrant. The cultivar G-Z2-M and G-SF-M were located in the same quadrant, but also were not separated well. The result of bi-plot PC1 *versus* PC3 was similar to the bi-plot PC1 *versus* PC2. This suggested that these cultivars may have the similar anthocyanin biosynthesis, and their relative was closer than the rest of the cultivars. Figure 3B is a scatter plot showing the distribution of these grape cultivars, according to PC 1 *versus* PC 2 and PC 1 *versus* PC 3 at harvest. In the scatter plot PC 1 *versus* PC 2, G-ZY-H and G-ZH-H was distanced well, although both of them were located at the same quadrant. However, the cultivars G-SH-H, G-Z2-H, G-SF-H, G-SY-H was closely assembled in the fourth quadrant. In the scatter plot PC 1 *versus* PC 3, the cultivar G-SY-H was separated into the first quadrant and was close to G-Z1-H. However, the results were similar to the scatter plot of PC 1 *versus* PC 2.

Comparing the scatter plot at maturation with other plots of the same harvest, it can be seen from Table 1 that the anthocyanin profiles are influenced by the period of grape development. W-ZH wine was totally separated from other wines along PC 1 (Figure 3C). According to the PC1 and PC2, W-Z2 and W-SH were located at the top left side, while W-Z1 and W-SY were located at the bottom left. However, W-ZY was very close to W-SF, as were W-SH, W-Z1 and W-SY. When the samples were separated according to the PC1 and PC3, W-ZY and W-SF were enough separated into two different locations, but not all the wines could be differentiated applying the two principal components plot (PC1 *versus* PC2 or PC1 *versus* PC3). This is the case of the groups: W-ZY/W-SF/W-SH, W-SY/W-Z1 (PC1 *versus* PC2); W-SF/W-SY/W-Z2, W-ZY/W-Z1/W-SH (PC1 *versus* PC3).

## 4. Conclusion

In this experiment, 17 anthocyanins were identified from skins and wines of seven grape cultivars, including 11 anthocyanin monoglucosides (five pyranoanthocyanin monoglucosides and one acylated pyranoanthocyanin monoglucoside) and six anthocyanin diglucosides. 15 anthocyanins were identified from their wines, including nine anthocyanin monoglucosides (four pyranoanthocyanin monoglucosides) and six anthocyanin diglucosides. 14 were identified anthocyanins from their skins, including eight anthocyanin monoglucosides (two pyranoanthocyanin monoglucosides and one acylated pyranoanthocyanin monoglucoside) and six anthocyanin diglucosides. In the skins of *V. amurensis grape* and its hybrids, the anthocyanins profiles are the anthocyanin monoglucosides, diglucosides and pyranoanthocyanins. The wines created from these produced the different anthocyanins distribution. Pelargonidin-3,5-*O*-diglucoside was detected in the *V. amurensis*, its hybrids and their wines, while the pyanoanthocyanins were also detected in the grape skin. These characters of anthocyanin profile of *V. amurensis* and its hybrids are helpful for differing the grape cultivars from other grape species (e.g., *V. vinifera*, *V. labrusca, V. rotundifolia*), and identifying the authenticity of red wines. The PCA results also suggested that the anthocyanin profiles are helpful to classify these cultivars of *V. amurensis*. In addition, the anthocyanin profile of *V. amurensis* is also important for the study of anthocyanin biological syntheses, because they include pyranoanthocyanins while excluding the non-acylated anthocyanins.

## Figures and Tables

**Table 1.** Anthocyanins in grape skins (mg/g dry weight) and wines (mg/L) of *Vitis amurensis* and its hybrids identified by HPLC-ESI-MS/MS.

**Peak No.**	**RT**	**Molecular and Product Ions (*m/z*)**	**Anthocyanins**	**G-SF**			**G-SH**			**G-SY**			**G-Z1**			**G-Z2**			**G-ZH**			**G-ZY**		

				**M**	**H**	**W**	**M**	**H**	**W**	**M**	**H**	**W**	**M**	**H**	**W**	**M**	**H**	**W**	**M**	**H**	**W**	**M**	**H**	**W**
**1**	4.39	627(M^+^) 465 303	Dp-3,5-diglu	0.971	1.001	104.947	1.647	1.658	141.346	0.222	0.592	33.805	0.364	1.273	39.142	1.401	1.538	82.849	0.477	0.991	429.317	1.919	2.582	132.893
**2**	6.69	611(M^+^) 449 287	Cy-3,5-diglu	0.021	0.081	162.987	0.805	0.877	120.936	0.465	0.641	321.699	0.693	1.952	237.122	0.181	0.195	101.334	0.227	0.509	340.570	0.587	0.595	72.141
**3**	7.88	641(M^+^) 479 317	Pt-3,5-diglu	0.865	0.985	16.289	1.603	1.612	178.779	0.675	0.681	10.593	0.363	1.197	89.413	0.806	1.518	94.896	-	0.493	227.028	1.122	1.225	49.037
**4**	8.53	465(M^+^) 303	Dp-3-glu	0.838	1.616	1.552	0.686	0.742	46.261	0.176	0.231	2.668	0.287	0.351	21.903	0.171	0.321	10.438	0.887	2.333	165.848	2.893	3.063	17.212
**5**	9.13	595(M^+^) 433 271	Pg-3,5-diglu	trace	trace	trace	trace	trace	trace	trace	trace	trace	trace	trace	trace	trace	trace	trace	trace	trace	trace	trace	trace	trace
**6**	12.09	625(M^+^) 463 301	Pn-3,5-diglu	0.097	0.285	233.255	0.565	0.612	83.824	1.165	1.059	221.071	1.654	2.256	287.448	0.474	0.497	87.052	1.415	1.142	234.246	0.245	0.536	113.569
**7**	12.89	449(M^+^) 287	Cy-3-glu	trace	trace	trace	trace	trace	trace	trace	trace	trace	trace	trace	trace	trace	trace	trace	0.013	0.011	25.351	trace	trace	trace
**8**	13.48	655(M^+^) 493 331	Mv-3,5-diglu	4.179	4.719	745.097	3.432	5.817	807.682	4.737	7.547	532.164	3.836	6.885	822.060	7.191	9.529	1040.209	1.532	2.343	680.975	3.606	4.259	767.377
**9**	16.49	479(M^+^) 317	Pt-3-glu	0.167	0.187	-	0.144	0.161	11.812	0.047	0.167	1.445	0.073	0.089	8.603	0.069	0.097	5.641	0.465	0.514	191.291	0.466	0.515	4.578
**10**	23.53	463(M^+^) 301	Pn-3-glu	0.005	0.015	5.652	0.083	0.105	-	0.019	0.025	5.652	0.019	0.023	1.119	0.009	0.016	61.024	0.032	0.035	45.203	0.032	0.035	-
**11**	25.61	493(M^+^) 331	Mv-3-glu	0.069	0.462	5.513	0.381	0.382	15.385	0.457	0.591	7.006	0.412	0.673	15.429	0.629	0.963	9.634	2.334	1.028	32.916	0.807	0.901	9.565
**12**	26.13	487(M^+^) 325	Pn-3-glu-4-ace	-	-	8.070	-	-	1.148	-	-	2.137	-	-	0.876	-	-	10.048	-	-	10.047	-	-	-
**13**	26.24	561(M^+^) 399	Mv-3-glu-4-py	-	-	8.097	-	-	-	-	-	1.769	-	-	-	-	-	6.121	-	-	6.120	-	-	2.165
**14**	27.26	517(M^+^) 355	Mv-3-glu-4-ace	-	-	0.624	-	-	0.189	-	-	-	-	-	-	-	-	-	-	-	0.229	-	-	0.209
**15**	28.44	531(M^+^) 369	Pn-3-glu-4-py	-	-	0.241	-	-	1.709	-	0.004	0.314	0.001	0.009	0.453	0.002	0.003	16.343	trace	trace	2.501	-	0.001	0.123
**16**	29.86	609(M^+^) 447	Mv-3-glu-4-vinylphen	-	-	-	-	-	-	-	-	-	-	-	-	-	-	-	-	-	-	0.005	0.002	-
**17**	30.09	651(M^+^) 447	Mv-3-Acglu-4-vinylphen	-	-	-	-	-	-	-	-	-	-	-	-	-	-	-	-	-	-	-	0.007	-

Shuang Feng (G-SF), Shuang Hong (G-SH), Shuang You (G-SY), Zuo Shan Yi (G-Z1), Zuo Shan Er (G-Z2), Zuo Hong Yi (G-ZH), Zuo You Hong (G-ZY). M: Maturation, H: harvest, W: wine, ‘-’ not detected, trace: content < 0.001 mg/g.

**Abbreviations:** glu: glucoside, diglu: diglucoside, Dp: Delphinidin, Pt: Petunidin, Mv: Malvidin, Pn: Peonidin, Cy: Cyanidin, Pg: Pelargonidin, ace: acetaldehyde, py: pyruvic acid, Acglu: acetylglucoside, vinylphen: vinylphenol.
